# Stem cell delivery to kidney *via* minimally invasive ultrasound-guided renal artery injection in mice

**DOI:** 10.1038/s41598-020-64417-2

**Published:** 2020-05-05

**Authors:** May Zaw Thin, Olumide Ogunlade, Joan Comenge, P. Stephen Patrick, Daniel J. Stuckey, Anna L. David, Mark F. Lythgoe, Paul Beard, Tammy L. Kalber

**Affiliations:** 1 UCL Centre for Advanced Biomedical Imaging, Division of Medicine, University College London, London, WC1E 6DD UK; 2 Department of Medical Physics & Biomedical Engineering, University College London, London, UK; 3 Institute of Integrative Biology, University of Liverpool, Liverpool, L69 7ZB UK; 4 UCL Institute for Women’s Health, London, WC1E 6BT UK

**Keywords:** Ultrasound, Mesenchymal stem cells

## Abstract

Cell-based therapies are promising treatments for various kidney diseases. However, the major hurdle in initiating therapeutic responses is the inefficiency of injection routes to deliver cells to the kidney parenchyma. Systemic injection, such as intravenous injection only delivers a small proportion of cells to the kidney. Whereas direct delivery, such as renal artery injection requires surgical procedures. A minimally invasive renal artery injection was therefore developed to enhance cell delivery to kidney. In this study, luciferase expressing human adipocyte derived stem cells (ADSC) were labelled with gold nanorods (GNR) and injected into the renal artery using ultrasound guidance. The ADSCs were tracked using bioluminescence and photoacoustic imaging serially over 7 days. Imaging confirmed that the majority of signal was within the kidney, indicative of successful injection and that the cells remained viable for 3 days. Histology showed co-localization of GNRs with ADSC staining throughout the kidney with no indication of injury caused by injection. These findings demonstrate that ultrasound-guided renal artery injection is feasible in mice and can successfully deliver a large proportion of cells which are retained within the kidney for 3 days. Therefore, the techniques developed here will be useful for optimising cell therapy in kidney diseases.

## Introduction

Over the past few years, stem cell-based regenerative therapy has been vastly studied as an alternative treatment for various kidney diseases. Mesenchymal stem cells or stromal cells (MSCs) have been used to repair kidney damage due to their ability to release a variety of active biological factors including growth factors such as insulin-like factor 1 (IGF-1)^1^, vascular endothelial growth factor (VEGF)^2^, and other immune system signalling molecules, such as transforming growth factor-β (TGF- β)^3^. Through these signalling cascades, MSCs are able to recruit and promote repair processes during acute kidney injury (AKI). Therefore, MSC-based therapy has been tested in several AKI mouse models and has been used to treat patients with AKI in three clinical trials^4^.

Although many studies have demonstrated the efficacy of stem cell therapy in kidney diseases, the engraftment of stem cells within the kidney parenchyma as a routine clinical treatment is hindered by the inefficient delivery and retention of stem cells after transplantation. Intravenous (IV), intra-arterial and intraparenchymal injections have been tested in many mouse models of AKI to deliver MSCs to kidney. Although IV injection is a relatively easy and non-invasive method of administration, the main problem is the pulmonary entrapment of cells directly after injection which thereby reduces engraftment within the kidney^5^. Several studies have shown that systemic intra-arterial delivery which bypasses the lung can provide increased cell retention in kidney^6^. However, evidence of vascular occlusion after intra-arterial injection has been reported which raises safety concerns^7^. A study by *Alfarano et al*., directly injected cells into the renal parenchyma which resulted in reduced kidney fibrosis in a rat model of renal ischemia-reperfusion^8^ but this approach is difficult to implement in a clinical setting due to the risk of additional injury. Similarly, the delivery of MSCs directly into the renal artery of an injured kidney has been shown to increase cell engraftment in preclinical studies^9,10^. However, this method is invasive and is usually conducted at the end of the open abdominal surgery to induce renal ischemia. Therefore, the development of a less invasive and more efficient injection route is urgently required to improve stem cell delivery to preclinical kidney injury models.

In addition to establishing the optimal route for stem cell delivery, tracking of transplanted cells and verifying their viability within the target organ is equally important in determining therapeutic efficacy of the cell therapy. Nuclear imaging using ^111^In-oxine labelling has been used to track stem cells in a mouse model of renal ischemia-reperfusion injury^11^. However, due to its low spatial resolution, it lacks detailed information on cell localisation. Iron oxide particles are one of the most widely used MRI-based cell tracking agents for locating transplanted cells in kidney^9,12,13^. Although MRI can provide excellent spatial resolution, iron oxide particles produce negative contrast which makes it difficult to quantify the number of cells in kidney. In addition, the presence of iron oxide particles will interfere with potential functional MRI assessments utilized for kidney diseases such as blood flow measurements^14^. Despite many studies having explored the applicability of various fluorescent probes, such as near infrared dyes for stem cell tracking in kidney^15,16^, the utility of optical imaging is limited owing to light scattering which reduces the spatial resolution and penetration depth.

Compared to current imaging modalities, photoacoustic (PA) imaging is a relatively new but rapidly expanding non-invasive imaging modality. PA imaging is based on the detection of laser generated ultrasound waves and provides images based on the optical absorption properties of chromophores such as haemoglobin. Since acoustic waves are not scattered as much as light photons, PA imaging avoids the depth and spatial resolution limitations of purely optical imaging techniques that arise due to strong light scattering in tissue. A previous study has demonstrated the great potential of PA as a cell tracking tool by using a tyrosinase-based genetic reporter^17^. However, the production of melanin from this reporter gene can be cytotoxic to some cell types and therefore may not be suitable for stem cell tracking^18^. Therefore, to use PA imaging in tracking cell therapies, the contrast agents need to have minimal cytotoxicity with little interference in the cell’s biological functions.

Since gold is known to be non-cytotoxic and inert in biological environment, gold nanoparticles (GNP) have been extensively used for *in vitro* cell imaging^19^. Some studies have reported that *in vivo* therapeutic efficacy of GNP labelled cells were unaffected in a rat model of neuropsychiatric disorders^20^ and a mouse model of subcutaneous tumours^21^. Gold nanoparticles, such as gold nanorods (GNRs) are also promising PA contrast agents due to their unique optical properties which allow optical absorption in the near infrared (NIR) window, where optical absorption of tissue is minimal. This is because the peak optical absorption wavelength of GNRs, due to surface plasmon resonance, can be tuned by modifying the shape of the GNR ^22^. By absorbing strongly in the NIR window, the PA detection sensitivity of GNRs can be increased. Moreover, unlike iron oxide nanoparticles, GNRs do not impede any functional assessment of the target organ by MRI. For these reasons, GNRs have been successfully applied as stem cell tracking agents for PA imaging^23^. As many studies have showed the advantages of PA imaging in detecting kidney disease such as ischemic kidney damage^24^, early kidney injury^25^, Adriamycin-induced nephropathy^26^ and polycystic kidney diseases^27^, tracking GNR-labelled MSCs in kidney with PA imaging will provide accurate cell localisation together with the structural and functional status of the kidney.

In this study, a novel non-surgical ultrasound-guided renal artery injection was developed to improve stem cell delivery to the kidney without the need for open abdominal surgery. In addition, a dual bioluminescence imaging (BLI) and PA imaging approach was applied in this study by labelling luciferase and green fluorescent protein (GFP) expressing ADSCs with silica coated GNRs. The silica-coating preserves the optical properties of GNR by preventing plasmon coupling thereby increasing photoacoustic sensitivity^23^. This allowed for the visualisation of cell viability (BLI) with cell localisation (PA) within the kidney serially over time after ultrasound-guided renal artery injection.

## Result

### Intracellular uptake of GNRs and their effect on cell proliferation and differentiation potential

**Figure 1:**
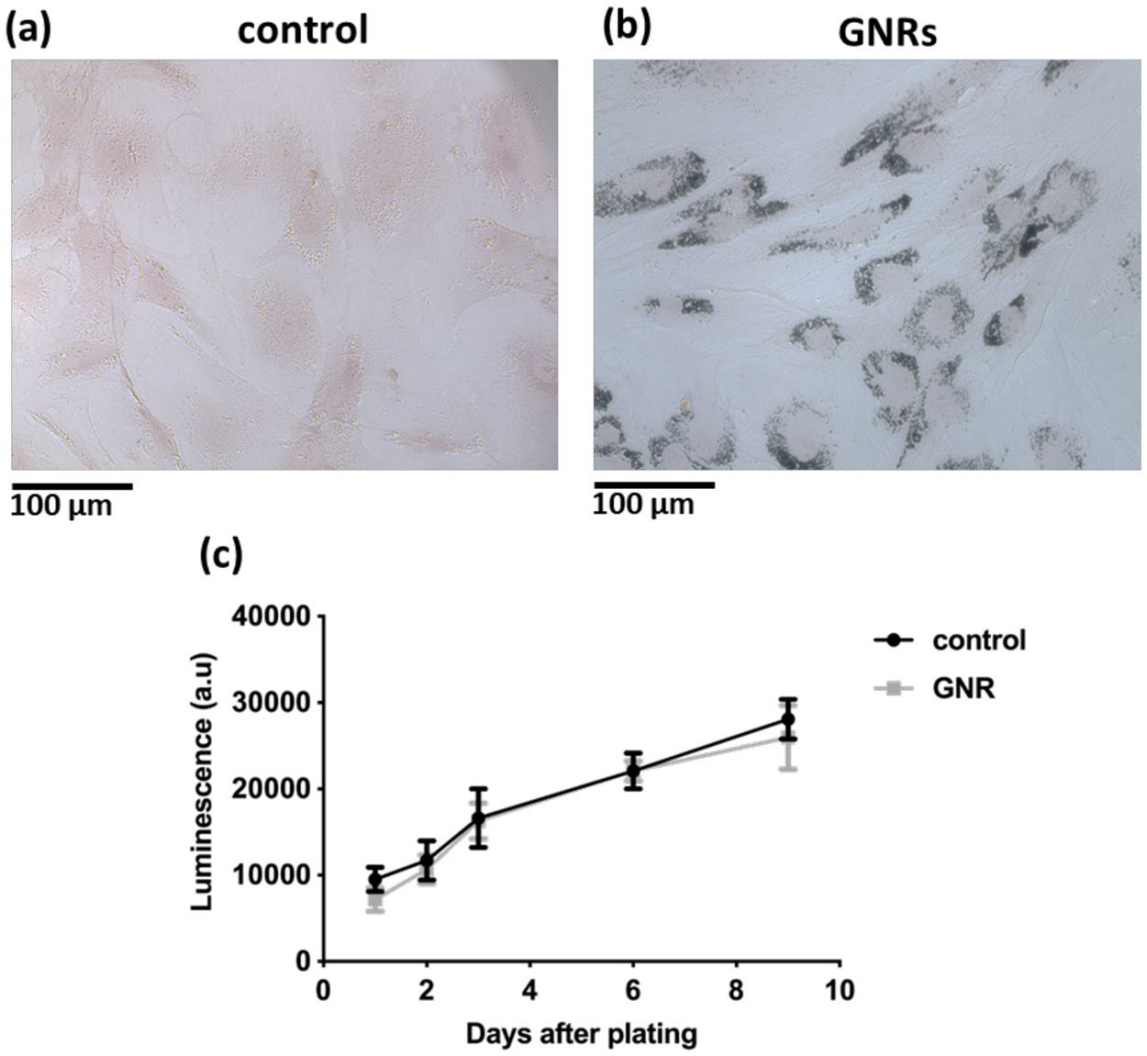
Intracellular uptake of GNRs in ADSCs and the effect on cell proliferation. (**a**,**b**) Silver enhancement staining of ADSCs treated with GNRs showing the dark aggregates inside the cytoplasm compared to untreated control (scale bar = 100 µm). (**c**) Luciferase-based cell proliferation assay at different time points showing no significant difference between BLI signals (luminescence) of control and GNR labelled ADSCs (data are shown as mean ± SD, n = 3).

**Figure 2:**
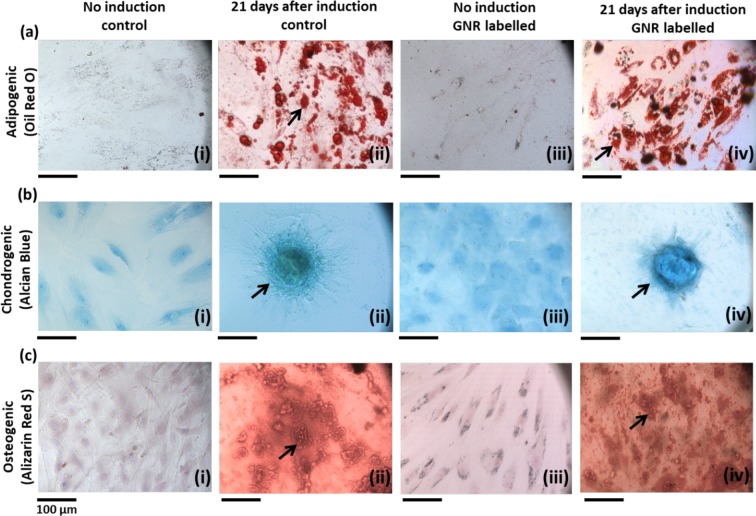
Tri-lineage differentiation of control ADSCs and GNR labelled ADSCs. (**a**) Oil red o staining for adipogenic differentiation which displays the red coloured oil droplets (indicated by arrows in (ii & iv)). (**b**) Alcian blue staining for chondrogenic differentiation which displays the blue coloured proteoglycans (indicated by arrows in (ii & iv)). (**c**) Alizarin red s staining for osteogenic differentiation which displays the red coloured calcium deposits (indicated by arrows in (ii & iv)). Scale bar = 100 µm.

After 24 hours incubation with silica coated GNRs, internalisation of GNRs by ADSCs was visualised under a light microscope using silver enhancement staining which showed the presence of dark aggregates inside the cytoplasm of ADSCs compared to control (Fig. 1a,b). The effect on cell proliferation of ADSCs after GNR labelling was studied by measuring bioluminescence emission as a surrogate measurement of cell proliferation. There was no difference in signal intensity between control and GNR labelled groups which showed an increase in signal from day 1 to day 3 after plating which remained stable throughout the study (Fig. 1c). The effect on tri-lineage (adipogenic, chondrogenic and osteogenic) differentiation potential of ADSCs after GNR labelling was assessed by performing differentiation assays (Fig. 2). The results showed both the control and GNR labelled cells could differentiate towards tri-lineages at a similar rate. These results indicate that GNR labelling has no adverse effect on cell proliferation and differentiation potential of ADSCs.

### Stem cell delivery to kidney via ultrasound-guided renal artery injection

**Figure 3:**
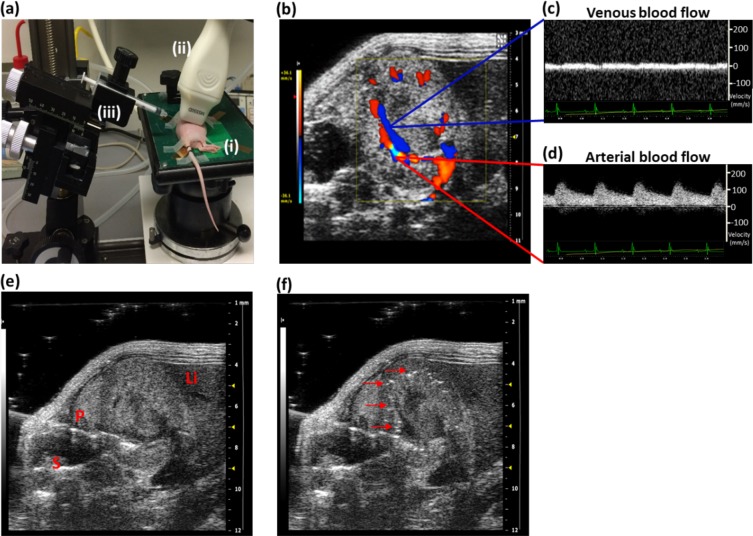
Non-surgical ultrasound-guided renal artery injection. (**a**) The animal was positioned in left lateral position on the platform (i) with the ultrasound transducer (ii) on the right lower quadrant of the abdomen and the syringe in injection mount (iii) facing towards the paravertebral muscle of the animal. (**b**) Colour Doppler image of the kidney showing renal blood supply (**c**,**d**) Pulsed wave Doppler images identifying the venous and arterial flow velocity pattern. (**e**) The needle was pierced through the skin and the vertebral muscle of the animal to penetrate into the right renal artery (labels: P = paravertebral muscle, S = spine, Li = liver). (**f**) The successful injection was visualised by the presence of hyperechogenic contrast around renal cortex (indicated by arrows).

Ultrasound-guided renal artery injection was performed using 6–8-week-old female nude mice. Under anaesthesia, mice were positioned in the left lateral position on the ultrasound platform with the injection mount positioned towards the spine of the animal (Fig. 3a). A colour Doppler image of the right kidney was acquired to visualise the renal blood vessels (Fig. 3b). The flow velocity waveforms of renal artery and vein were identified using a pulsed wave Doppler sample gate (Fig. 3c,d).

2 ×10^5^ GNR labelled ADSCs were suspended in 100 μl of 0.6% w/v alginate solution and injected slowly into the renal artery. In order to avoid surrounding organ damage, the needle was passed through the paravertebral muscle and then into the renal artery (Fig. 3e, Supplementary Video 1). Successful injection was visualised by the presence of hyperechogenic contrast from the alginate solution around the renal cortex (Fig. 3f, Supplementary Video 2). The needle was slowly withdrawn from the body after 15 seconds delay to prevent washing out backwards. Colour Doppler imaging showed renal artery flow returned within 20 seconds after injection (Supplementary Video 3).

The entire protocol took approximately 15 minutes and the heart rate, body temperature and respiration rate were monitored throughout the procedure. No significant weight loss or sign of major trauma was observed as monitored by body weight and colour of the urine throughout the study. The injection technique was optimised using 13 animals and the success rate of injection was 70% (9 out of 13 animals).

### Assessment of the cell viability and cell localisation following renal artery injection

To assess the viability and localisation of GNR labelled ADSCs within the kidney, the mice were imaged with BLI and PA imaging serially for 7 days after ultrasound-guided right renal artery injection.

**Figure 4:**
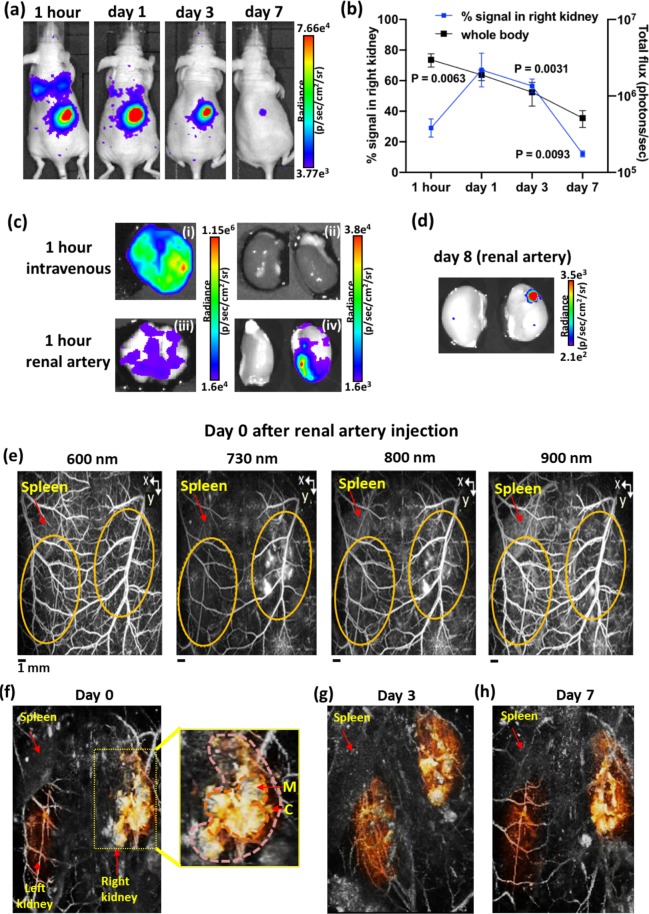
BLI and PA images of GNR labelled ADSC in right kidney at different time points after renal artery injection. (**a**) BLI images at 1 hour, days 1, 3 and 7 after injection showing the highest signal within the right kidney and some signal from the lungs. (**b**) The percentage BLI signal in right kidney relative to whole body (1 hour vs days 1, 3 & 7 using unpaired two-tailed t test) and BLI signal from the whole body decreased over time (photons/sec in log scale). (**c**) *Ex vivo* BLI images showing high signal from the lungs (i) and no signal from the kidneys (ii) at 1 hour after IV injection and low signal from the lungs (iii) and high signal from the right kidney (iv) at 1 hour after renal artery injection. (**d**) *Ex vivo* BLI images at day 8 after renal artery injection showing reduced signal from the right kidney. (**e**) PA images acquired at different wavelength showing the higher PA contrast in right kidney (indicated by yellow dotted lines) than the surrounding renal blood vessels at 730 nm using the spleen as anatomical landmark (images are presented as MIP). (**f**–**h**) 3D volume-rendered PA images at days 0, 3 and 7 after injection showing the widespread PA contrast in right kidney including cortex (C) and medulla (M), compared to left kidney (the kidneys were manually segmented and false coloured). Data are shown as mean ± SD, n = 3.

At 1 hour after injection, the highest BLI signal was coming from the right kidney (Fig. 4a) with some signal from the lungs. The percentage BLI signal in right kidney (relative to whole body BLI signal) was 29 ± 6% (Fig. 4b). BLI images at days 1 and 3 after injection showed the persistence of the signal within the right kidney (Fig. 4a) and the percentage BLI signal was significantly higher than 1-hour post injection (P = 0.0063 & P = 0.0031, Fig. 4b). At day 7 after injection, BLI images showed a reduced signal within the right kidney (Fig. 4a**)** and the percentage BLI signal was significantly lower than 1-hour post injection (P = 0.0093, Fig. 4b). Observation across the rest of the body showed the BLI signal intensity from the lungs and the whole body decreased over time (Fig. 4b) and no sign of cell migration to other organs was seen. To validate and compare the cell distribution pattern in the lungs and the kidneys, *ex vivo* BLI was performed at 1 hour and day 8 after renal artery injection and at 1 hour after intravenous injection of the same number (2 × 10^5^) of cells. The signal intensity from the lungs at 1 hour after IV injection was higher than after renal artery injection and no BLI signal was detected in both kidneys (Fig. 4c). *Ex vivo* BLI images at 1 hour after renal artery injection are consistent with *in vivo* BLI suggesting that the majority of cells were delivered to the right kidney and remained viable up to day 8 (Fig. 4d).

Immediately after BLI, PA images of both kidneys were acquired at 3-5 hours after injection (termed day 0) using a range of wavelength from 600 to 900 nm (Fig. 4e,f). PA images appeared to show a stronger signal within the right kidney compared to the left kidney at all wavelengths. However, images acquired at 730 nm showed the highest PA contrast in the right kidney when compared to the surrounding renal blood vessels (Fig. 4e). This is consistent with the absorption peak of the GNRs (750 nm) which indicated the presence of GNR labelled cells within the right kidney. In addition, this signal was detected throughout the right kidney including cortex and medulla in 3D volume rendered PA images (Fig. 4f) and the signal intensity remained stable for 3 days (Fig. 4g). At day 7 after injection, high signal intensity was still present in PA images (Fig. 4h) although a reduced BLI signal was detected in the right kidney.

### Histological analysis

**Figure 5:**
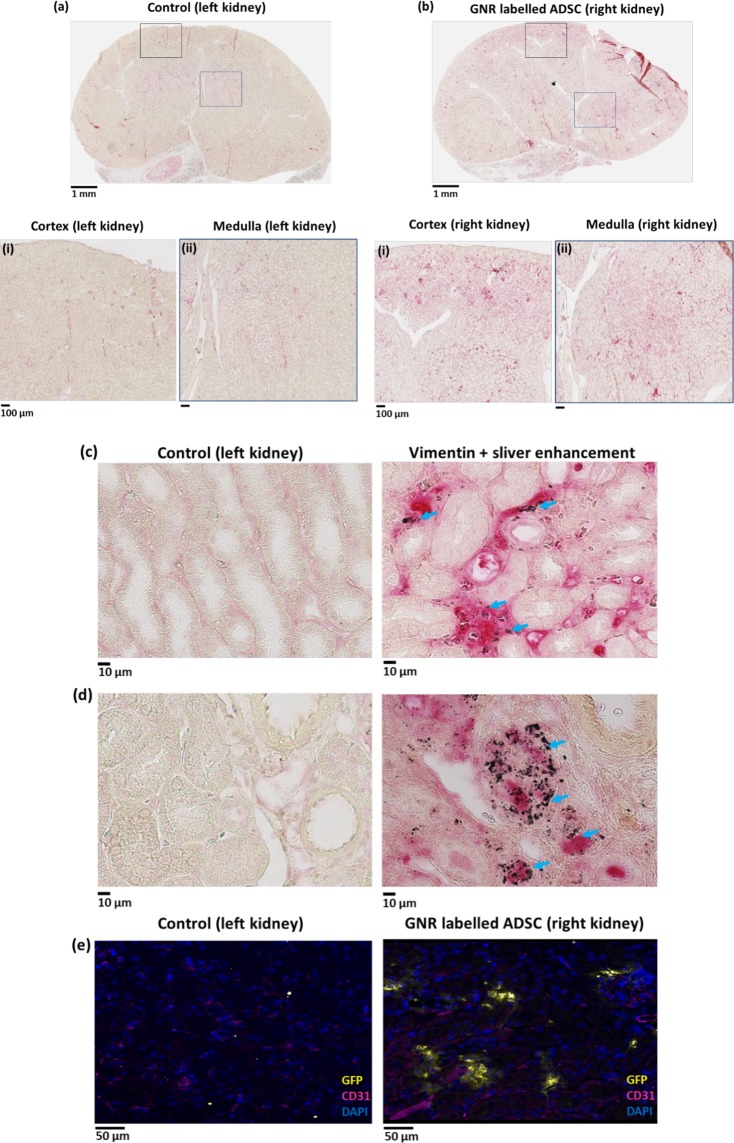
Histological analysis of GNR labelled ADSCs uptake in kidney at day 8 after right renal artery injection. (**a**,**b**) Vimentin (ADSC) staining of control (left kidney) and injected right kidney sections showing the widespread red staining in the cortex and medulla of right kidney compared to the control. Positive vimentin (ADSC) and silver enhancement staining of right kidney tissue sections showing the presence of dual labelled cells within the renal (**c**) capillary bed and (**d**) parenchyma compared to control (indicated by blue arrows, scale bar = 10 µm). (**e**) Immunofluorescence images of the control and injected kidney sections showing the overlapping of GFP staining (ADSC) with CD31 staining (renal microvasculature, indicated by white arrows, scale bar = 50 µm).

In order to validate the presence of GNR-labelled ADSCs in the right kidney after renal artery injection, the kidney sections from day 8 after injection were stained with human specific vimentin (ADSCs) and silver enhancement (GNRs) using the left kidney for control tissue sections (Fig. 5a). Histological images showed widespread vimentin staining throughout the right kidney including cortex and medulla confirming the presence of ADSCs in the right kidney (Fig. 5b). Higher magnification images showed that areas positive for vimentin staining were also positive for silver enhancement staining (Fig. 5c,d), confirming the retention of GNRs within ADSCs. In addition, the dual labelled cells were detected within the renal capillary bed (Fig. 5c) and renal parenchyma (Fig. 5d) compared to the left kidney tissue section. The similar finding was seen in immunofluorescence images showing positive GFP staining in the right kidney (Fig. 5e). There was no evidence of renal pathology in particular, no signs of trauma or haemorrhage resulting from the injection and no malignant tumour formation that could potentially result from ADSC engraftment.

## Discussion

The aims of this study were to improve stem cell delivery in preclinical kidney models by developing a minimally-invasive ultrasound-guided renal artery injection and to subsequently assess the viability and localisation of delivered cells by serial BLI and PA imaging over seven days.

The intracellular uptake of GNRs by ADSCs was studied *in vitro* and the results showed that GNRs were internalised inside the cytoplasm. These results are consistent with studies carried out by *Comenge et al., 2016* who showed the entrapment of the same formulation of GNRs within the cytoplasmic organelles of MSCs^23^. The results from *in vitro* luciferase and differentiation assays demonstrated that GNR labelling had no effect on ADSC proliferation and tri-lineage differentiation potential indicating that GNRs have no detectable cytotoxic effect on ADSCs which is also consistent with the findings from *Comenge et al., 2016*.

Ultrasound-guided renal artery injection of GNR-labelled cells demonstrated that this method is feasible, fast and less invasive than the routine renal artery injection using open abdomen surgical techniques. The main strength of the ultrasound-guided technique is a faster recovery and healing time with fewer complications such as infections in immunocompromised mice. Besides, this method can be adapted as a clinical application compared to invasive intra-renal parenchymal injection and will be advantageous for studies where repeated cell deliveries are required. During initial studies, the spillage of cells into the abdominal cavity was observed when the cells were re-suspended in PBS (results not shown). Since the alginate solution is known to be viscous^28^ and commonly used in ultrasound contrast agents^29^, the re-suspension medium was changed to 0.6% alginate solution to prevent spillage and to improve the accuracy of the injection by taking advantage of the ultrasound contrast provided by alginate. In addition, the high temporal resolution of ultrasound allows for optimisation of the injection techniques.

The distribution of GNR labelled cells in the kidney after injection was assessed by using BLI and PA imaging. At 1 hour after injection, although the majority of the signal (29 ± 6%) was detected in the injected right kidney, there was some signal from the lungs in BLI images. Interestingly, BLI signal from the lungs decreased from day 1 after injection and was not detectable at day 7 while the percentage BLI signal in right kidney significantly increased at days 1 and 3 after injection. In comparison, many studies have reported the persistence of BLI signal within the lungs for 7 days after IV injection^6,30^. Unlike the persistent cell trapping after IV injection, it is most likely that a certain percentage of cells flowed into the renal vein after passing through the renal capillary bed and then to the lungs via venous drainage.

It has been well documented that the majority of stem cells are trapped within the pulmonary microvasculature following IV injection with limited distribution to distal organs^31–33^. To bypass the cell accumulation in lungs, alternative injection routes, such as intra-cardiac injection have been investigated in several studies^34–36^. However, these methods have failed to deliver the majority of cells to the kidney. In this study, we have shown that the majority of the GNR labelled cells were retained within the kidney and remained viable for 3 days after ultrasound-guided renal artery injection. In addition, the widespread cell distribution across the kidney, including the cortex and medulla was detected in PA images and further confirmed by histological analysis. These results highlight the advantages of using PA imaging which can provide the high spatial resolution of cell localisation in the kidney.

Although consistent findings were seen between BLI and PA images at early time points, discrepancies were observed at day 7 after injection. Since BLI is a reporter gene-based cell tracking method that requires ATP, the signal generation depends on cell survival, while in PA imaging, the presence of signal only represents the existence of the direct labelling agent, GNR, not the cell itself. Therefore, if GNRs remain within the cell after cell death then the PA signal remains even when BLI decreases. These results consequently suggest that only a small number of cells remain viable 7 days after injection but the majority of dead cells are retained within the kidney. Nonetheless, these studies were done in mice with no underlying kidney disease and therefore the length of cell viability could be altered in disease state.

PA images were taken at different wavelengths to discern signal from the vasculature (600 nm) from that of GNR labelled cells (730 nm). Although the highest PA signal within the right kidney was at 730 nm without spectral unmixing, it is unclear if some underlying signal came from haemorrhage. However, histological findings at day 8 after injection confirmed the widespread presence of GNR labelled ADSCs throughout the kidney with no evidence of haemorrhage or injury derived from the injection of ADSCs. Mice showed no evidence of wound haematoma or macroscopic haematuria at any point. In addition, no biosafety risks such as vascular occlusion or *in vivo* toxicity due to GNRs was observed during the study. Although histology showed no signs of tumour formation, there are still safety concerns regarding MSC-based therapies in kidney diseases. In addition, the maldifferentiation of MSC into glomerular adipocytes and sclerosis after renal artery injection have been reported in a rat model of renal failure^37^. Therefore, more long-term studies are required to investigate the fate of MSCs and the clearance of GNRs in recipient organs after transplantation.

In summary, the results presented in this study demonstrated the successful development of a minimally invasive ultrasound-guided renal artery injection which can improve stem cell delivery to the kidney. However, the success rate of injection may vary depending on the architecture of renal vasculature of diseased models. For example, the success rate may be lower in animal models of renal artery stenosis compared to polycystic kidney disease with renal vessel dilatation^27^. Nevertheless, the findings from this study displayed the benefits of using a more refined and effective injection technique over invasive and inefficient delivery routes. In addition, the advantages of combining a reporter gene (luciferase, BLI) with a direct cell labelling method (GNR, PA) have been highlighted in this study. The new techniques which have been developed in this study can be applied to track other stem cell types such as kidney derived stem or progenitor cells. The information acquired from this study can therefore be applied to the optimisation of cell therapies in various rodent models of kidney diseases.

## Materials and methods

All chemical materials were from Sigma-Aldrich (Dorset, UK), unless otherwise stated.

### Silica coated gold nanorods synthesis

All experiments were performed using silica coated GNRs which were kindly provided by Dr Joan Comenge, Institute of Integrative Biology, University of Liverpool. Detailed synthesis and characterisation of GNRs were published in *Comenge et al., 2016*. Synthesis of GNRs was performed using a previously published protocol^38^. Silica coating of GNRs was carried out by adjusting a previously described method^39^. Briefly, cetyl trimethyl ammonium bromide (CTAB) capped GNRs was purified following two cycles of centrifugation. Methoxy polyethylene glycol thiol (mPEG-SH, 0.1 mM, MW = 5000 Da) was added to GNR solution. After 24 h under agitation, GNR pellet (7.5 mM) was obtained by centrifugation and then resuspended in ethanol. Silica condensation onto GNRs was performed by agitation for 2 h in a silica growth solution (0.2 M ammonium hydroxide, 0.5 mM atomic gold, 1.2 mM tetraethyl orthosilicate, 10.55 M water). Incubation and agitation steps were performed twice following two successive additions of tetraethyl orthosilicate. The core size of GNR was 21 ± 3 nm in width and 49 ± 5 nm in length with silica shell thickness of 34 ± 2 nm. The absorption peak of GNR was 756 nm with the optical density of 0.74. Silica coated GNRs were kept in ethanol solution for storage and they were resuspended in water just before cell labelling.

### Cell culture

All experiments were performed using human ADSCs which were kindly provided by Dr Michelle Griffin, UCL Plastic and Reconstructive Surgery Department. ADSCs were transduced with a lentiviral vector using plasmid pSEW^40^ to express green fluorescence protein (GFP) and firefly luciferase^41^ under the control of the Friend murine leukaemia virus FB29 promoter. The successful transduction was confirmed by GFP expression using a fluorescence microscope (EVOS FL Auto cell imaging system, ThermoFisher Scientific, Massachusetts, USA) and the transduction efficiency was 42.56 ± 7.43%. The transduced ADSCs were grown in T175 flasks (Fisher Scientific, Loughborough, UK) in DMEM-F12, supplemented with 10% fetal calf serum (FCS, Invitrogen, Paisley, UK) in a humidified incubator at 37 °C with 95% air and 5% CO_2_. Cells were grown to 80% confluence before being trypsinised, centrifuged for pelleting at 300 g, counted and then plated for *in vitro* experiments or GNR labelling.

### Lentivirus production

Lentivirus encoding firefly luciferase and GFP was produced in HEK 293 T cells using calcium phosphate precipitation protocol adapted from that described by *Tiscornia et al*.^42^, using the transfer plasmid pSEW-Flagx3-FLuc-2A-GFP (which was a kind gift from Dr Martin Pule, UCL Cancer Institute), together with packaging plasmids, Gag-pol (pCMV-R8.74; Addgene Plasmid # 22036) and VSV-G (pMD2.G; Addgene Plasmid # 12259). To improve viral titres, sodium butyrate (1 mM) was added to the media, 24 hours prior to lentiviral harvest^43^. Lentivirus was harvested into ADSC culture medium (DMEM-F12, supplemented with 10% fetal calf serum), passed through a 20 µm syringe filter, and added directly to ADSCs for transduction. After 24 hours ADSCs were changed into fresh media.

### ADSC labelling with GNRs

3 × 10^5^ ADSCs were seeded in a T25 flask with 5 ml of DMEM-F12 supplemented with 10% FCS and left to attach overnight. The next day, cells were incubated with GNRs at a concentration of 0.04235 mgAu/ml (80% medium + 20% GNRs in water) for 24 hours. After washing 3 times with PBS, the labelled ADSCs were trypsinised, centrifuged for pelleting at 300 g, counted and then plated for in vitro studies or re-suspended in 0.6% alginate solution for *in vivo* cell injection.

### Silver enhancement staining

To visualise intracellular GNR uptake by ADSCs, silver enhancement staining was performed according to manufacturer’s instructions (silver enhancer kit, Sigma-Aldrich, St Louis, USA). GNR labelled ADSCs were plated in 24-well plates at a concentration of 0.22 × 10^5^ per well in triplicates and left to attach overnight. Next, the well plates were washed 3 times with PBS and the cells were fixed in 4% PFA for 30 minutes at room temperature. The cells were then washed 2 times with PBS followed by staining with silver enhancer mixture for 10 minutes at 20 °C. The cells were then washed 2 times with distilled water and fixed with 2.5% sodium thiosulfate solution for 3 minutes. Fixative was removed by washing 3 times with distilled water and the cells were counterstained with 1% Nuclear Fast Red and then imaged with EVOS FL Auto cell imaging system.

### *In vitro* luciferase assay

For luciferase-based cell proliferation assay, control ADSCs and GNR labelled ADSCs were plated in 96-well plates at a concentration of 5 × 10^3^ per well in triplicates. *In vitro* luciferase assay was performed using a Varioskan LUX multimode microplate reader (ThermoFisher Scientific, Waltham, MA USA) at days 1, 2, 3, 6 and 9 after plating. The measurements were acquired immediately after adding 300 μg/ml of D-luciferin and the results were presented as luminescence.

### Differentiation assay

To investigate the effect of GNR labelling on the differentiation potential of ADSCs, a differentiation assay was performed as previously described^44^. Control and GNR labelled ADSCs were plated in 24-well plates (Corning) at a concentration of 0.22 × 10^5^ per well in triplicates. When both control and labelled cells reached 90-100% confluency, the regular culture media was removed and differentiation media (adipogenic, chondrogenic or osteogenic) was added to appropriate wells and was changed every three days. Undifferentiated wells received regular media. After 3 weeks, staining was performed for each differentiation and the cells were imaged using the EVOS FL Auto cell imaging system.

### Adipogenic differentiation

Adipogenic differentiation was induced with DMEM-F12 medium containing 10% FCS, 1% penicillin/streptomycin, 10 ng/ml insulin, 1 μM dexamethasone, 500 µM 3-isobutyl-1-methylxanthine and 1 mM rosiglitazone. After 3 weeks, cells were fixed in 10% formalin for 30 minutes, washed with deionised water and then washed again with 60% isopropanol for 5 minutes prior to staining with Oil Red O working solution for 10 minutes. After staining, cells were washed several times with tap water.

### Chondrogenic differentiation

Chondrogenic differentiation was induced with DMEM-F12 medium containing 10% FCS, 1% penicillin/streptomycin, 0.1 μM dexamethasone, 50 μg/ml ascorbate, 10 ng/ml transforming growth factor (TGF) β1 (Life technologies, Paisley, UK) and 10 ng/ml insulin, transferrin, selenium. After 3 weeks, cells were fixed in 4% paraformaldehyde (PFA) for 30 minutes and then washed with deionised water. Then rinsed with 0.1 M HCl for 5 minutes and stained with Alcian Blue staining (1% in 0.1 M HCl) for 30 minutes. After staining the cells were washed with tap water 3 times.

### Osteogenic differentiation

Osteogenic differentiation was induced with DMEM-F12 medium containing 10% FCS, 1% penicillin/streptomycin, 0.1 μM dexamethasone, 10 mM β-glycerophosphate and 100 μg/ml ascorbate. After 3 weeks, cells were fixed in ice-cold 70% ethanol for 1 hour and washed with deionised water. Then the cells were stained with Alizarin Red staining (1% in deionised water, pH 4.1–4.3). After staining, cells were washed with tap water 3 times.

### *In vivo* studies

All animal studies were approved by the University College London Biological Services Ethical Review Committee and licensed under the UK Home Office regulations and the Guidance for the Operation of Animals (Scientific Procedures) Act 1986 (Home Office, London, United Kingdom). All mice were 6-8-week-old female CD-1 nude mice (Charles River Laboratories, UK) and all *in vivo* imaging experiments were performed under isoflurane anaesthesia (1.5%–2.5% isoflurane in oxygen 1.5–2 l/min). The signs of injury resulting from injection and the general wellbeing of the animals were observed throughout the study by monitoring body weight and colour of urine.

### *In vivo* cell injections

Mice were imaged and injected using a VEVO 2100 ultrasound imaging system (VEVO 2100, FUJIFILM VisualSonics, Canada) with a Visualsonics ultrasound platform and injection rig. Mice were secured in the left lateral position with tape and their heart rate, body temperature and respiration rate were monitored throughout the procedure. The injection mount with syringe holder was positioned towards the paravertebral muscle of the animal. Ultrasound gel was applied to the right lower quadrant of the abdomen and the right kidney was located using the liver as anatomical reference. Renal blood vessels were identified using a colour Doppler mode. In order to identify the arterial and venous flow velocity waveforms, a pulsed wave Doppler sample gate was placed within the vessels at the appropriate angle relative to flow direction.

2 × 10^5^ GNR labelled ADSCs (in 100 μl of 0.6% alginate solution) were injected into the right renal artery of mice (n = 3) using a 1 mL syringe with a 29 g needle. Total ultrasound injection and imaging took approximately 15 minutes, mice were then imaged serially with BLI and PA for 7 days. Injection optimisation studies were performed in 13 mice but no serial imaging was carried out in these animals.

### *In vivo* imaging

#### BLI

*In vivo* BLI was performed at 1 hour, days 1, 3 and 7 after renal artery injection using an IVIS Lumina (PerkinElmer, USA). Mice were injected intraperitoneally with 75 mg/kg D-luciferin (Promega) in 200 µl of PBS. Sequential BLI images were acquired 5 minutes after luciferin injection using auto exposure time with 0.5 minutes delay between two consecutive acquisitions. A rectangular region of interest (ROI) was placed over the whole body on the first image and subsequently pasted over every new image acquired until all ROIs reach their maximum intensity. The total signal in the ROI was quantified as total flux (photons/s) by using Living Image software version 4.5 (PerkinElmer). To calculate the percentage of BLI signal in right kidney, a circular ROI was placed over the right kidney and the signal from the right kidney ROI was divided by the whole-body signal and presented as % signal in right kidney. Representative images were presented using radiance (the number of photons per second that leave a square centimetre of tissue and radiate into a solid angle of one steradian (sr) = p/sec/cm^2^/sr) as colour scale by utilizing the same software.

#### PA imaging

Directly after BLI imaging, mice were anaesthetised and PA scans were acquired at 3-5 hours after injection (termed day 0), and then at days 3 and 7 using a planar PA scanner (built in the Department of Medical Physics and Biomedical Engineering, UCL) based on a Fabry-Pérot (FP) polymer film ultrasound sensor and a tunable oscillator laser system (Quanta Ray Pro-270/premiScan; Newport Spectra Physics/GWU Lasertechnik). The PA images of right and left kidneys were acquired as previous described in *Ogunlade et al*.^27^. In brief, the animals were placed in a supine position and a small amount of ultrasound gel was applied to the lower back of the animals. Short (7 ns) laser pulses were used to illuminate the tissue at a repetition rate of 50Hz. . Photoacoustic signals were generated and detected by the FP ultrasound sensor using four different wavelengths between 600-900 nm (600, 730, 800 and 900 nm) to differentiate between signal derived from haemoglobin and that from GNR (however, spectral unmixing was not performed in this instance). An integrated heater and thermal chamber with temperature set to 34 °C was used during the imaging to maintain core body temperature. The images were displayed as MIPs and 3D volume-rendered images. False colouring, manual segmentation and extracting of the vasculature architecture was performed using Amira (FEI Visualization Sciences).

### *Ex vivo* BLI imaging

*Ex vivo* BLI was performed at 1 hour and day 8 after renal artery injection and 1 hour after IV injection of 2 × 10^5^ GNR labelled ADSCs in 100 µl of PBS. Under anaesthesia, mice were injected intraperitoneally with 75 mg/kg D-luciferin. At 10 minutes after injection, mice were sacrificed and the organs of interest were excised quickly and images were acquired using 5 minutes exposure time and binning 8.

### Histology and immunohistochemistry (IHC)

At day 8 after renal artery injection, right and left kidneys were excised, fixed in 10% neutral buffered formalin and embedded in paraffin. Human specific vimentin (CONFIRM™ anti-Vimentin (V9) primary mouse monoclonal antibody, Roche) staining was performed on 5 μm kidney tissue sections to detect the presence of ADSCs using Ventana Discovery XT instrument and Ventana Red detection kit. For pre-treatment, Ventana CC1, equivalent to EDTA buffer, was used. To demonstrate the presence of GNRs, IHC stained sections were stained with silver enhancement staining (silver enhancer kit, Sigma-Aldrich) according to manufacturer’s instructions. The sections were scanned with Nanozoomer slide scanner (Hamamatsu Photonics, Japan). The images were viewed with NanoZoomer Digital Pathology software (NDP Version 2.7.25). For immunofluorescence, the deparaffinised and rehydrated sections were microwaved for 15 min in antigen retrieval solution (citrate pH 6, Sigma-Aldrich) and then blocked for 1 h in blocking solution (1% Bovine serum albumin and 5% FCS). Primary antibodies, goat polyclonal anti-GFP for detection of ADSC (Abcam, Cambridge, UK) and rabbit polyclonal anti CD31 (Abcam) antibodies for detection of renal vasculature, were applied overnight at 4 °C. Secondary antibodies, donkey polyclonal anti-goat Alexa Fluor 546-conjugated (Abcam) and donkey polyclonal anti-rabbit Alexa Fluor 647-conjugated (Abcam) antibodies were applied for 1 h at room temperature. Nuclei were labelled with 4′-6-diamidino-2-phenylindole (DAPI, Sigma-Aldrich). Images were acquired using a Zeiss LSM-780 inverted confocal microscope.

### Statistical analysis

All *in vitro* experiments were repeated at least 3 times with 3 triplicates.

Statistical analysis was performed with GraphPad Prism version 6.01. Data were presented as mean ± standard deviation (SD). Unpaired two-tailed t test was conducted for the assessment of statistical significance.

## Supplementary information

Supplementary video 1.

Supplementary video 2.

Supplementary video 3.
