# Supplementary figures and images for: Stem cell delivery to kidney via minimally invasive ultrasound-guided renal artery injection in mice
Source: Sci Rep. 2020 May 5;10:7514. doi: 10.1038/s41598-020-64417-2 (PMC7200714; doi:10.1038/s41598-020-64417-2)

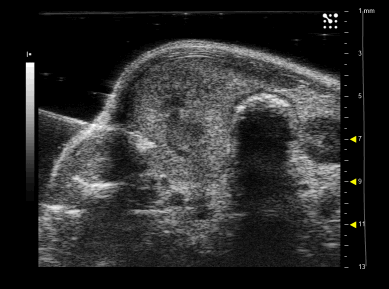

Supplement: Supplementary file 1 — Supplementary video 1. [file 41598_2020_64417_MOESM1_ESM.gif]

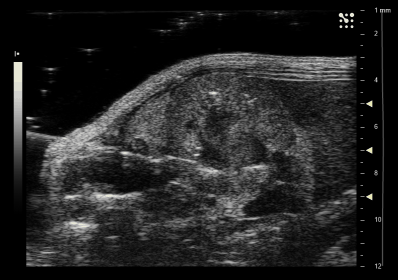

Supplement: Supplementary file 2 — Supplementary video 2. [file 41598_2020_64417_MOESM2_ESM.gif]

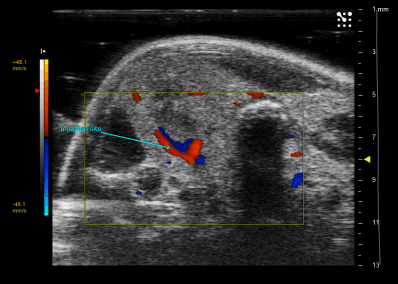

Supplement: Supplementary file 3 — Supplementary video 3. [file 41598_2020_64417_MOESM3_ESM.gif]
